# Review: Effect of Gut Microbiota and Its Metabolite SCFAs on Radiation-Induced Intestinal Injury

**DOI:** 10.3389/fcimb.2021.577236

**Published:** 2021-07-09

**Authors:** Yangyang Li, Yiming Zhang, Kongxi Wei, Jinpeng He, Nan Ding, Junrui Hua, Ting Zhou, Fan Niu, Gucheng Zhou, Tongfan Shi, Liying Zhang, Yongqi Liu

**Affiliations:** ^1^ Provincial-Level Key Laboratory for Molecular Medicine of Major Diseases and The Prevention and Treatment With Traditional Chinese Medicine Research in Gansu Colleges and Universities, Gansu University of Chinese Medicine, Lanzhou, China; ^2^ Key Laboratory of Space Radiobiology of Gansu Province & Key Laboratory of Heavy Ion Radiation Biology and Medicine of Chinese Academy of Sciences Institute of Modern Physics, Chinese Academy of Sciences, Lanzhou, China; ^3^ Gansu Institute of Cardiovascular Diseases, Lanzhou, China; ^4^ Key Laboratory of Dunhuang Medicine and Transformation at Provincial and Ministerial Level, Lanzhou, China

**Keywords:** radiotherapy, gut microbiota, dysbiosis, metabolites, SCFAs, radiation-induced intestinal injury

## Abstract

Gut microbiota is regarded as the second human genome and forgotten organ, which is symbiotic with the human host and cannot live and exist alone. The gut microbiota performs multiple physiological functions and plays a pivotal role in host health and intestinal homeostasis. However, the gut microbiota can always be affected by various factors and among them, it is radiotherapy that results in gut microbiota ^1^ ^2^dysbiosis and it is often embodied in a decrease in the abundance and diversity of gut microbiota, an increase in harmful bacteria and a decrease in beneficial bacteria, thereby affecting many disease states, especially intestine diseases. Furthermore, gut microbiota can produce a variety of metabolites, among which short-chain fatty acids (SCFAs) are one of the most abundant and important metabolites. More importantly, SCFAs can be identified as second messengers to promote signal transduction and affect the occurrence and development of diseases. Radiotherapy can lead to the alterations of SCFAs-producing bacteria and cause changes in SCFAs, which is associated with a variety of diseases such as radiation-induced intestinal injury. However, the specific mechanism of its occurrence is not yet clear. Therefore, this review intends to emphasize the alterations of gut microbiota after radiotherapy and highlight the alterations of SCFAs-producing bacteria and SCFAs to explore the mechanisms of radiation-induced intestinal injury from the perspective of gut microbiota and its metabolite SCFAs.

## Introduction

Gut microbiota, second genome of the human body, as the genes that it carries are about 100 times more than the human genome (Singh et al., 2017; Chassaing and Cascales, 2018; Knauf et al., 2019). It is estimated that about 10^14^ microorganisms reside in the human intestinal tract, weighing 1-2 kg, which includes bacteria, archaea, fungi, and viruses. These microorganisms are commensal with human host, 90% of the symbiotic microorganisms in the digestive tract (Singh et al., 2017). Although there are individual differences in the composition of gut microbiota (Lynch Susan and Pedersen, 2016; Bouter Kristien et al., 2017; Budden Kurtis et al., 2017), it plays an important role in maintaining homeostasis and internal environment stability through its metabolites. The gut microbiota can produce a variety of small molecules and metabolites, among which short chain fatty acids (SCFAs) can connect intestinal flora and host to play a key physiological role (Xing et al., 2020). They can serve as messengers, with the ability to alter the gut microbiota, thereby affecting various disease states (Yadav et al., 2018). Various factors affect the composition and function of gut microbiota, influencing the physiological and pathological state of the host. But the influence has great differences (Ursell et al., 2014; Serino, 2019). Among them, radiotherapy can have an important impact on gut microbiota.

Radiotherapy is one of the main treatments for cancer patients in which more than 50% of those patients receive it, but its side effects cannot be ignored (Citrin, 2017; De Ruysscher et al., 2019). Recently, radiation-induced intestinal injury has attracted more and more attention. The reason is that the intestine is extremely sensitive to ionizing radiation, especially in the radiotherapy of abdominal and pelvic malignant tumors, the healthy intestine is inevitably exposed to the radiation (Hauer-Jensen et al., 2014; Kumagai et al., 2018). The current research on the mechanism of radiation-induced intestinal injury has the following explanation: intestinal epithelial injury, intestinal microvascular changes, immune mechanism, neuro-immune interaction, gut microbiota and many other factors (Hauer-Jensen et al., 2014). It is gut microbiota and its metabolites (especially SCFAs) play significant roles in radiation-induced intestinal injury. Radiotherapy leads to abnormal intestinal motility, which promotes the colonization of intestinal flora in the gastrointestinal tract, and these two are also important factors for the development of severe radiation enteropathy (Husebye et al., 1995). Crawford et al. have studied irradiated germ-free mouse with γ-rays, indicating a link between gut microbiota and radiation enteritis (Crawford and Gordon, 2005). Extensive evidence already showed that the diversity of intestinal flora and the concentration of SCFAs decreased in radiation-induced intestinal injury (Ferreira et al., 2014). Therefore, in this review, we will outline current knowledge about the changes in gut microbiota and SCFAs after radiotherapy. We will also highlight the specific mechanism associated with SCFAs on radiation-induced intestinal injury, thus providing a feasible basis for clinical diagnosis and treatment.

## Radiotherapy Leads to Gut Microbiota Dysbiosis and Changes in SCFA

### Gut Microbiota Dysbiosis

#### The Effect of Radiotherapy on the Gut Microbiota

Radiotherapy can cause damage to multiple organ systems, and the degree of damage is usually dose-dependent (Kiang and Olabisi, 2019). Dysbiosis in the gut is one of the main damage outcomes, and it is described in **Table 1**. ROSOFF has found that when exposed to whole body radiation reaching a lethal dose, it can cause death. After radiation, intestinal bacteria is isolated, so it is believed that the suppression of intestinal flora has an important effect on the ability to recover after fatal radiation (Rosoff, 1963). Hou et al. have studied the effects of intestinal bacterial depletion on mice receiving 12Gy single-dose whole-body irradiation (TBI). They found that the use of broad-spectrum antibiotics that disrupt commensal bacteria to be harmful to mammals receiving lethal TBI, indicating that the gut microbiota plays a pivotal role in the body (Hou et al., 2007). Husebye et al. have revealed the relationship between intestinal motility and gastrointestinal flora, in which abnormal motility was linked to the colonization of Gram-negative bacteria in the gastrointestinal tract. Meanwhile, they have pointed out that abnormal intestinal motility and Gram-negative bacilli in the proximal small intestine are important factors in the pathogenesis of severe late radiation enteropathy (Husebye et al., 1995). In particular, radiotherapy for malignant tumors in the abdominal area may interfere with the colonization resistance of the endogenous intestinal flora. The destruction of colonization resistance of intestinal flora is the main pathophysiological mechanism of radiation enteritis, which is also a common and serious complication of patients after receiving radiotherapy (Visich and Yeo, 2010). Lam et al. have studied the adult male Wistar rats received single or multiple whole body irradiations of 10.0 Gy and 18.0 Gy. Microarray and quantitative PCR (polymerase chain reaction) analysis were used to determine the composition of fecal microbiota. Radiation exposure biomarkers include the 16S rRNA levels of 12 members of *Bacteroidales*, *Lactobacillaceae*, and *Streptococcaceae* increased after radiation exposure, the levels of 98 *Clostridiaceae* and *Peptostreptococcaceae* remained unchanged, and the levels of 47 *Clostridiaceae* members decreased. The characterization of the bacterial flora confirms that the intestinal flora can be used as a new biomarker for radiation exposure (Lam et al., 2012). Nam et al. have conducted a prospective observation study of intestinal flora in gynecological cancer patients undergoing pelvic radiotherapy. In this study, 454 pyrosequencing was used to study the overall composition and changes of the intestinal microflora of cancer patients undergoing radiotherapy. The results showed that the intestinal microbial composition of cancer patients was significantly different from that of healthy people. Radiotherapy resulted in a significant reduction in the number and abundance of intestinal flora. Specifically after treatment, the number of *phyla Firmicutes* decreased and the number of *Fusobacteria* increased. In addition, pelvic radiotherapy also affects the highly individual-specific intestinal flora of cancer patients, thereby gradually reshaping the composition of the intestinal microflora. However, the specific classification of radiotherapy affecting the intestinal flora is still uncertain (Nam et al., 2013).The gut microbiota plays an important role in regulating immune homeostasis in the host. Patients undergoing radiotherapy resulting in cytotoxicity showed significant changes in the intestinal flora, the most common of which were the decrease of *Bifidobacterium*, *Clostridium cluster XIVa* and *Faecalibacterium prausnitzii* and increase of *Enterobacteriaceae* and *Bacteroides*. These flora changes have promoted the development of gastrointestinal mucositis, mainly by changing the intestinal barrier function, innate immunity and intestinal repair mechanisms (Touchefeu et al., 2014). Kim et al. have characterized the large intestine and small intestine flora of mice after γ-ray irradiation by Illumina MiSeq high-throughput sequencing platform and bacterial 16S rRNA gene analysis, and have found that the abundance and diversity of intestinal flora change a lot after irradiation. At the phylum level, radiation causes a decrease in the phyla *Firmicute*s and *Actinobacteria* in the large and small intestines, while radiation increases the number of *Bacteroidetes* in the large intestine and the number of *Proteobacteria* in the small intestine. Furthermore, several genera in the microflora of the large and small intestine have been identified at the genera level. The most abundant bacterial genera in the small intestine are *Turicibacter* and *Corynebacte rium*, and the subclass of this organ is *Alistipes*. In general, at the genus level, the bacterial diversity of small intestine is much smaller than that of large intestine. In the large intestine, irradiation increases the proportion of *Alistipes*, *Lactobacillus* and *Akkermansia*, but decreases the proportion of *Barnesiella*, *Prevotella*, *Bacteroides*, *Oscillibacter*, *Pseudoflavonifractor* and *Mucispirillum*. Additionally, in the small intestine, the radiation caused significant changes in the five genera of *Turicibacter*, *Corynebacterium*, *Alistipes*, *Lactobacillus* and *Muciprillum*. Among them, the increase of *Corynebacterium* abundance and the decrease of *Alisipes* abundance are the most obvious (Kim et al., 2015). Goudarzi et al. have used 16S rRNA sequencing and metabolomics to determine the fecal metabolomics characteristics of x-ray irradiated mice. 16S rRNA sequencing results showed that the intestinal flora changed significantly after irradiation. After 5 and 12Gy X-ray irradiation, it was found that the abundance of common bacteria in *Lactobacillaceae* and *Staphylococcacea* increased, while the abundance of bacteria in *Lachnospiraceae*, *Ruminococcaceae* and *Clostridiaceae* decreased. Metabolomics data showed that the metabolites of intestinal flora changed significantly such as pipecolic acid, glutaconic acid, urobilinogen and homogentisic acid. In addition, there were significant changes in bile acids such as taurocholic acid and 12-ketodeoxycholic acid (Goudarzi et al., 2016).

**Table 1:** Radiotherapy causes gut microbiota dysbiosis.

Model	Sample type	Sequencing Method	Gut microbiota dysbiosis	Reference
Male Wistar rats	Fecal samples	Microarray (16S rRNA) and quantitative PCR analyses	Increase: 12 members of *Bacteroidales*, *Lactobacillaceae*, and *Streptococcaceae*	(Lam et al., 2012)
			Decrease: the levels of 47 *Clostridiaceae* members	
nine gynecologic cancer patients	Fecal samples	Pyrosequencing of bacterial 16S rRNA fragments	Increase: *Fusobacteria*	(Nam et al., 2013)
			Decrease: the number and abundance, *phyla Firmicutes*	
C57BL/6 mice	the contents of the small and large intestines	Illumina MiSeq high-throughput sequencing and bacterial 16S rRNA	Increase: *Bacteroidetes* and *Firmicutes*	(Kim et al., 2015)
			Decrease: *phyla Firmicute*s and *Actinobacteria*	
C57BL/6J mice	Fecal samples	16S rRNA sequencing and metabolomics	Increase: *Firmicutes*, common bacteria in *Lactobacillaceae* and *Staphylococcacea*	(Goudarzi et al., 2016)
			Decrease : *Bacteroidetes*, *Lachnospiraceae, Ruminococcaceae* and *Clostridiaceae*	
Gottingen minipigs (GMP) and Chinese rhesus macaques	Fecal samples	Illumina MiSeq sequencing and 16S rRNA amplicon	Increase:intracellular symbionts (*Elusimicrobia* in GMP, *Spirochaetes* in macaques), *Firmicutes* in minipigs	(Carbonero et al., 2018; Carbonero et al., 2019)
			Decrease: *Bacteroidetes* and *Proteobacteria*	
137 bank voles *Myodes glareolus*	Fecal samples	amplicon sequencing of bacterial 16S rRNA genes	Increase: *Bacteroides*	(Lavrinienko et al., 2018)
Male BALB/c mice	Fecal samples	high-throughput sequencing of 16S rRNA	Increase : *Clostridium*, *Helicobacter* and *Oscilibacter*	(Liu et al., 2019)
			Decrease: *Bacteroides* and *Barnesiella*	
Patients with and without radiation enteropathy	Fecal samples, intestinal mucosa samples	Metataxonomics (16S rRNA gene) and imputed metataxonomics (Piphillin)	Increase: *Clostridium IV*, *Roseburia*, and *Phascolarctobacterium*	(Reis Ferreira et al., 2019)
			Decrease: bacterial diversity	
18 cervical cancer patients	Fecal samples	16S rRNA sequencing using the Illumina HiSeq platform	Increase: β‐diversity, *Proteobacteria* and *Gammaproteobacteria*	(Wang et al., 2019)
			Decrease: α‐diversity, *Bacteroides*.	

#### Radiosensitivity of Gut Microbiota of Different Species

Different species have different radiosensitivity. Carbonero et al. have studied the changes in intestinal flora of Gottingen minipigs (GMP) and rhesus macaques in acute radiation syndrome. They have found that although GMP and rhesus macaques have different intestinal microflora distributions, radiation had a similar effect at the phylum level, resulting in a decrease in *Bacteroidetes* and an increase in *Firmicutes* in both models. Irradiation significantly reduced the abundance of the main *Bacteroidetes genus* (*Bacteroides* for GMP, *Prevotella* for macaques). Intracellular symbionts (*Elusimicrobia* in GMP, *Spirochaetes* in macaques) continue to increase after irradiation, indicating that they are potential biomarkers of intestinal damage. The abundance of *Prevotella*, *Lactobacillus*, *Clostridium XIVa*, *Oscillibacter* and *Elusimicrobium*/*Treponema* is significantly related to the radiation intensity (Carbonero et al., 2019). They have also compared the bacterial population changes of the two species in the acute radiation syndrome after bioequivalent dose irradiation, and have found that there is a general increase of intracellular symbionts in both models, indicating that these findings are universal after radiation. It is worth noting that the opposite dynamics are observed in the main door, with *Firmicutes* increased in minipigs and *Bacteroidetes* and *Proteobacteria* decreased but *Bacteroidetes* are rich in macaques set. The size of the miniature pigs and changes in the affected species were more extensive than those observed in rhesus macaques, indicating that different species have different sensitivity to radiation (Carbonero et al., 2018). Lavrinienko et al. have studied the effect of environmental radionuclides on the intestinal flora of bank voles Myodes glareolus. It has been found that exposure to high levels of environmental radionuclides had no significant effect on the intestinal flora abundance, but it was related to almost twice the increase of *Bacteroides* compared with *Firmicutes : Bacteroidetes* ratio (Lavrinienko et al., 2018). Liu et al. have studied and compared the composition of intestinal flora in mice exposed to low dose ionizing radiation (LDR). Male BALB/c mice were exposed to low dose of Co^60^ radiation and fecal samples collected before and after irradiation were used for high-throughput sequencing of 16S rRNA gene sequence amplicons. They have observed substantial changes in the composition of the intestinal flora, including alpha and beta diversity, in mice exposed to LDR compared to the control group without radiation. They have also found that the abundance of *Clostridium*, *Helicobacter* and *Oscilibacter* after radiation increased in a time-dependent manner, while the abundance of *Bacteroides* and *Barnesiella* showed a time-dependent decline. In addition, these changes in the gut microflora are accompanied by changes in the abundance of multiple metabolites (Liu et al., 2019).

#### The Relationship Between Gut Microbiota Dysbiosis and Radiation-Induced Intestinal Injury

There is a close relationship between gut microbiota dysbiosis and intestinal injury after radiotherapy. Reis Ferreira et al. have reported that the largest clinical study to date to explore the relationship between microbiota and acute and late radiation enteropathy. It has been found that the alteration of microbiota is associated with early and late radiation enteropathy and has clinical significance for the risk assessment, prevention and treatment of radiation side effects. Dynamically, a correlation between low bacterial diversity and early and late radiation enteropathy has also been observed. Higher *Clostridium IV*, *Roseburia*, and *Phascolarctobacterium* counts were significantly associated with radiation enteropathy(RE). In radiation-induced bowel disease, homeostatic intestinal mucosal cytokines associated with intestinal flora regulation and intestinal wall maintenance are significantly reduced (Reis Ferreira et al., 2019). Wang et al. have characterized the intestinal flora of radioactive enteritis caused by pelvic radiotherapy, and used bacterial epithelial co-culture to evaluate the epithelial inflammatory response. It has been found that intestinal microflora disorders is observed in patients with RE, which was characterized by a significant decrease in α diversity, but an increase in β diversity, a relatively high abundance of *Proteobacteria* and *Gammaproteobacteria*, and less abundance of *Bacteroides*. Metastat analysis further revealed unique microbiological characteristics associated with grades, such as the more abundant *Virgibacillus* and *Alcanivorax* in patients with mild enteritis. In addition, compared with the control flora, using bacterial-epithelial coculture, the RE-derived flora can induce epithelial inflammation and barrier dysfunction, and enhance the expression of TNF-α and IL-1β (Wang et al., 2019). Gerassy-Vainberg et al. have found that radiation-induced intestinal disorders increased the susceptibility of the intestine to RE and inflammation. These findings suggest that gut microbiota may be a key driver of RE process and provide the possibility to prevent or treat RE by targeting intestinal microbiota. In vivo experiments have shown that a small number of irradiated bacteria (rich in *Sutterella* in other bacteria) is enough to induce a higher susceptibility to intestinal inflammation, indicating that the reduction of bacterial diversity may lead to a short-term and long-term risk of enteropathy in patients (Gerassy-Vainberg et al., 2018; Sokol and Adolph Timon, 2018). Of course, radiotherapy can not only cause gut microbiota dysbiosis, but also change the corresponding metabolites, which will be explained in detail below.

### Changes of SCFAs Caused by Gut Microbiota Dysbiosis After Radiotherapy

The gut microbiota and its metabolites are in constant crosstalk with the host, and radiotherapy has been shown to have an important impact on gut microbiota and metabolites, especially SCFAs. Casero et al. have found in the study of space radiation (^16^O radiation) that the gut microbiota changes dramatically after irradiation, which tends to the decrease of normal gut microbiota and shifts towards the increase of opportunistic pathogenic bacteria, causing intestinal microecological imbalance. In addition, this study has also suggested that the change of the gut microbiota induces the change in microbial metabolism and metabolic function. This shows the relationship between gut microbiota and its metabolites after radiation (Casero et al., 2017). Morgan JL et al. have found that the dietary high iron and radiation reduced the total concentration of some main SCFAs, and the decline was most pronounced when increased dietary iron is in an interactive mode with radiation exposure. The changes of fecal SCFAs concentration after the increase of dietary iron or exposure to radiation may be caused by the alterations of bacterial composition and/or the changes of bacterial metabolism (Morgan et al., 2014). According to an independent study, dietary iron consumption and radiation therapy (4.3-5.4Gy) can inhibit gut microbiota to varying degrees, including known butyrate producing bacteria. It has been reported that iron supplementation in the diet of iron deficient animals can increase the concentration of SCFAs in the cecum and the proportion of butyrate-producing bacteria (Dostal et al., 2012). After irradiation, the flora *Firmicutes* decreased significantly, while *Verrucomicrobia* and *Bacteroidetes* increased, while dietary pectin restored the bacteria to the control level and can reverse the decrease in intestinal microbial diversity caused by radiation. In addition, the concentrations of SCFAs and its main products acetate, propionate, butyrate, etc. decreased significantly after irradiation. The SCFAs are usually produced by the gut microbiota, so in fact, the decrease or the disruption that is caused to the bacteria by the radiation is what is leading for the decrease in SCFA production (Kim et al., 2015; Wang et al., 2015).

SCFAs, one of the most abundant microbial metabolites in the intestine (Ley et al., 2006; Turnbaugh et al., 2006), are the main end products of bacterial fermentation and typical representatives of the mutual relation between humans and their bacterial symbionts (Gentile and Weir, 2018) which are reported to be beneficial to human health (Cook and Sellin, 1998). SCFAs are mainly composed of acetate, propionate and butyrate, these three account for more than 95% and the rest of the ingredients are iso-butyrate, valerate, iso-valerate, hexanoate and the proportions of these ingredients change with diet (Miller and Wolin, 1996; De Filippo et al., 2010; Salonen et al., 2014; Koh et al., 2016).The common SCFAs-producing intestinal flora are mainly anaerobic bacteria, including *Bacteroides*, *Bifidobacterium*, *Clostridia*, *Streptococcus*, etc. (Marchesi et al., 2016) Acetate, propionate and butyrate are produced by their respective gut microbiota through different metabolic pathways. Acetate is produced by many enteric bacteria, such as *Akkermansia muciniphila*, *Bacteroides* spp., *Bifidobacterium* spp., *Prevotella* spp., *Ruminococcus* spp.via pyruvate in acetyl-CoA pathway and *Blautia hydrogenotrophica*, *Clostridium* spp., *Streptococcus* spp.via Wood-Ljungdahl pathway (Ragsdale and Pierce, 2008; Louis et al., 2014; Koh et al., 2016; Bose et al., 2019). Propionate is mainly produced by *Bacteroidetes* spp., some *Firmicutes* such as *Phascolarc-tobacterium succinatutens, Dialister* spp., *Veillonella* spp.via succinate pathway. It is also produced by *Roseburia inulinivorans* and *Ruminococcus obeum*, *Proteobacteria via* acrylate pathway and produced by *Roseburia inulinivorans, Ruminococcus obeum, Salmonella enterica via* propanediol pathway (Louis et al., 2014; Reichardt et al., 2014; Koh et al., 2016). Butyrate is produced by *Faecalibacterium prausnitzii*,*Roseburia* spp., *Eubacterium rectale*, *Eubacterium hallii*, *Anaerostipes* spp. *via* butyryl-CoA:acetate CoA-transferase route, and produced by *Coprococcus catus via* phospho-transbutyrylase and butyrate kinase (Duncan et al., 2002; Hetzel et al., 2003; Louis et al., 2014; Vital et al., 2014; Koh et al., 2016). Radiotherapy caused a change in SCFAs-producing bacteria and a decrease in SCFAs. The different bacteria producing acetate, propionate and butyrate and the changes in SCFAs-producing bacteria and the decrease of SCFAs after radiotherapy are shown in **Table 2**.

**Table 2:** The production of SCFAs and changes after radiotherapy.

SCFAs	Producing bacteria	Biosynthetic pathway	Radiotherapy causes changes in SCFAs	Reference
Acetate	many enteric bacteria, such as *Akkermansia muciniphila*, *Bacteroides* spp., *Bifidobacterium* spp., *Prevotella* spp., *Ruminococcus* spp.	pyruvate in acetyl-CoA pathway	Decrease of SCFAs	(Louis et al., 2014; Koh et al., 2016)
	*Blautia hydrogenotrophica*, *Clostridium* spp., *Streptococcus* spp.	Wood-Ljungdahl Pathway		(Ragsdale and Pierce, 2008; Bose et al., 2019)
Propionate	*Bacteroidetes* spp., some *Firmicutes* such as *Phascolarc-tobacterium succinatutens, Dialister* spp., *Veillonella* spp., Roseburia spp., Firmicutes, Roseburia inulinivorans, Ruminococus spp., Cllostridium spp., Clostridiales bactrium, Eubacterium spp, Coprococcus spp., Dialister succinatiphilus, Phascolarctobaterium succinatutens, Akkermansia muciniphila	succinate pathway	Decrease of SCFAs	(Louis et al., 2014; Reichardt et al., 2014; Koh et al., 2016)
	some *Firmicutes* including *Roseburia inulinivorans* and *Ruminococcus obeum*, *Proteobacteria*, Clostridium spp., Clostridiales bacterium, Coproccus catus,	acrylate pathway		
	*Roseburia inulinivorans, Ruminococcus obeum Salmonella enterica*, Eubacterium halli, Clostridium sp.	propanediol pathway		
butyrate	*Faecalibacterium prausnitzii*, *Roseburia* spp., *Eubacterium rectale*, *Eubacterium hallii*, *Anaerostipes* spp.,	butyryl-CoA:acetate CoA-transferase route	Decrease of SCFAs	(Duncan et al., 2002; Hetzel et al., 2003; Louis et al., 2014; Vital et al., 2014; Koh et al., 2016)
	*Coprococcus catus*	phospho-transbutyrylase and butyrate kinase		

### Changes of SCFAs and Radiation-Induced Intestinal Injury

The intestine is highly sensitive to ionizing radiation, especially in the radiotherapy of abdominal and pelvic malignant tumors, the healthy intestine is inevitably exposed to the radiation, resulting in adverse consequences that are identified as radiation-induced intestinal injury (Hauer-Jensen et al., 2014; Kumagai et al., 2018). It has been reported that the prevalence of intestinal side effects caused by long-term radiation exceeds the combination of ulcerative colitis and Crohn’s disease (Hauer-Jensen et al., 2014). In general, there are acute and chronic types of radiation-induced intestinal injury according to the time of radiotherapy. Acute radiation-induced intestinal injury occurs within 3 months after radiotherapy, while chronic radiation-induced intestinal injury occurs more than 3 months after radiotherapy (Chater et al., 2019; Reis Ferreira et al., 2019). The main symptoms of acute radiation intestinal injury include abdominal pain, diarrhea, bleeding, fistula and perforation. The main manifestations of chronic radiation intestinal injury are intestinal obstruction, fibrosis and vascular sclerosis (Wang et al., 2006; Haydont and Vozenin-Brotons, 2007; Mangoni et al., 2017). Acute radiation-induced intestinal injury is characterized by an inflammatory response, while chronic radiation-induced intestinal injury is more prone to intestinal fibrosis, which is independent on inflammation and initially driven by TGF β1-mediated phenotype switch and transforms fibroblasts into collagen matrix and produces myofibroblasts (Yarnold and Brotons, 2010).

It is worth mentioning that there is a close correlation between changes in gut microbiota, SCFAs and radiation-induced intestinal injury. Radiotherapy reduces the ability of bacteria to produce SCFA and is associated with the appearance of symptoms of pelvic radiation disease (PRD) (Teo et al., 2015). A research has suggested that pelvic radiotherapy can cause acute small intestinal injury, and it can affect the nutritional status of the gut, so it is used to evaluate the morphological, nutritional, and functional changes after radiotherapy (Pía de la Maza et al., 2001). During pelvic radiotherapy, Wedlake et al. have proved that the SCFAs, fermentation products of soluble dietary fiber, promote the absorption of sodium and water, thus helping to relieve diarrhea after radiotherapy. In addition, increasing the intake of dietary fiber would increase the production of SCFAs and reduce the inflammatory process after radiotherapy, thus alleviating gastrointestinal toxicity after radiotherapy (Wedlake et al., 2017). Increasing fiber intake would increase SCFAs production and thereby reduce the inflammatory process (McOrist et al., 2011). In radiation-induced intestinal injury, SCFAs decreased, but dietary pectin could reverse and increase SCFAs so as to play a protective role in radiation-induced intestinal injury. Sureban et al. have reported that dietary pectin has a beneficial protective effect on the intestinal tract in mice model of acute intestinal injury after radiation by stimulating crypt proliferation (Sureban et al., 2015).

The gut microbiota is a crucial factor in radiation-induced intestinal injury, especially SCFAs which play a vital role in it. There is growing evidence that radiation causes significant changes in the gut microbiota of animals and humans. Multiple studies have indicated that the decline of bacterial diversity is consistently related to radiation-induced intestinal injury (Mosca et al., 2016; Cui et al., 2017; Castaño-Rodríguez et al., 2018). In the patients with acute severe diarrhea after pelvic radiotherapy, Manichanh et al. found that microbial diversity has changed a lot, and it was speculated that the change could be attributed to the colonization of different microorganisms (Manichanh et al., 2008; Ferreira et al., 2014; Wu et al., 2019). In recent years, it has been found that the level of SCFAs can indirectly respond to the regulation of gut microbiota. They all reduce intestinal inflammation by reducing the production of chemokines or adhesion molecules (Husted et al., 2017). In addition, studies have shown that the gut flora of patients with chronic radiation enteritis (CRE) is characterized by an increase in the number of *Gammaproteobacteria*, *Bacilli* and *Negativicutes* which are common in intestinal malnutrition, intestinal inflammation and radiation-induced intestinal injury (Lupp et al., 2007; Litvak et al., 2017). SCFAs are the main energy source of colon cells, and the use of SCFAs may be weaken in chronic radiation proctitis. The principle is that in the case of hemophilia induced by radiation, the related atrophy of mucosa may interfere with mitochondrial fatty acid oxidation, and SCFA supplementation in the form of enema can overcome this defect and improve the energy supply to colon cells (Denton et al., 2002; van de Wetering et al., 2016).

SCFAs play an important role in relieving intestinal injury induced by radiotherapy. In the study in mice, metabolomics revealed treatment with SCFAs(sodium acetate sodium butyrate or sodium propionate), especially propionate caused long-term radioprotection, mitigation of hematopoietic and gastrointestinal syndromes, and a reduction in proinflammatory responses, which is mediated by the attenuation of DNA damage and the release of reactive oxygen species in hematopoietic and gastrointestinal tissues (Guo et al., 2020). Preoperative radiotherapy may adversely affect certain mechanical and histological aspects of colonic anastomotic healing, but rectal irrigation with SCFAs may promote anastomotic healing (Terzi et al., 2004). SCFAs (such as acetate, propionate, and butyrate) play a protective role in in chemotherapy- or radiation-induced intestinal inflammation. Therefore, SCFAs have the certain therapeutic potential (Tian et al., 2020). Morever, the present study suggested that butyrate can enhance the efficacy of radiotherapy while protecting normal mucosa, thereby minimizing the toxicity caused by radiotherapy. The reason is that butyrate inhibits the proliferation of three-dimensional colorectal cancer organoids and enhances the radiation-induced cell death in colorectal cancer organoids through FOXO3A.However, butyrate does not increase radiation-induced cell death after irradiation of normal organoids (Park et al., 2020). In vitro experimental studies proved that sodium butyrate and valproic acid can enhance the radiosensitivity of human thyroid cancer cells (Perona et al., 2018).

## Mechanism of SCFAs in Radiation-Induced Intestinal Injury

Metabolites are often considered to function primarily as a “fuel” or energy source, or as the basis of a metabolic pathway. However, key metabolites (SCFAs are one of them) can also be regarded as extracellular signaling molecules, as they bind to receptors to activate a second messenger, thereby promoting downstream signal transduction (Husted et al., 2017).With regard to SCFAs, the two most studied mechanisms are: activation of G protein coupled receptor (GPCRs) [GPR41,GPR43 and GPR109A] and inhibition of histone deacetylase (HDAC). These characteristics of SCFAs affect their immunomodulatory potential, i.e. to maintain the anti-inflammatory/pro-inflammatory balance. SCFAs not only play a local role in the intestinal tract colonized by symbiotic bacteria, but also affects the intestinal immune cells and regulates the immune response through a variety of protein-inflammatory complexes (Ratajczak et al., 2019).

Due to rapid epithelial turnover, both the small and large intestine cause acute or chronic radiation damage to the intestine, especially when exposed to 45 Gy or higher doses of radiation (Gecse Krisztina, 2018). Radiation-induced intestine injury is pathologically similar to inflammatory bowel disease, and high-fiber intervention proved to be effective. The cohort study found that SCFAs producing bacteria were significantly correlated with the progression of radiation-induced intestinal injury, and the association is not random. Ionizing radiation can induce the release of proinflammatory factors such as IL-1β, TNFα and NF-κB (Somosy et al., 2002; François et al., 2013). SCFAs have anti-inflammatory activity and can inhibit the expression of these proinflammatory cytokines (Segain et al., 2000; Tedelind et al., 2007; Ferreira et al., 2014). SCFAs exerts anti-inflammatory activity by suppressing NF-κB and STAT1 activation (Postler and Ghosh, 2017; Spiljar et al., 2017; Belizário et al., 2018; Feng et al., 2018). A large number of studies have shown that butyrate can inhibit the NF-κB signaling pathway by rescuing the redox mechanism and controlling the reactive oxygen species that mediate the activation of NF-κB. In addition, butyrate exerts anti-inflammatory effects by activating PPAR-γ highly expressed in colon epithelial cells and inhibiting IFN-γ signaling (Liu et al., 2018).Butyrate can also inhibit HDACs and thus decrease the activation of their downstream signaling pathways VEGF and STAT3. Through anti-inflammation. Moreover, butyrate inhibits pro-inflammatory IL-6 and IL-17 which affect STAT3 and NF-κB signaling pathways (Chen et al., 2019). Furthermore, it has been confirmed that SCFAs exert anti-tumor activity through multiple signaling pathways, such as Wnt/β-catenin, PI3K/Akt/mTOR, MAPK (p38, JNK and ERK1/2), EGFR/Kras/Braf (Mármol et al., 2017; Feng et al., 2018; Afrin et al., 2020). NF-κB activation is also found in colorectal cancer and colitis-related tumors, which is responsible for the production of a variety of proinflammatory mediators and cytokines, such as the prostaglandin gene E2 (PGE2), inducible nitric oxide synthase (iNOS), COX-2, TNF-α, IL-6 and IL-1β,which in turn play a key role in the development of colorectal cancer (Karki et al., 2017; Ferrer-Mayorga et al., 2019).

**Figure 1:**
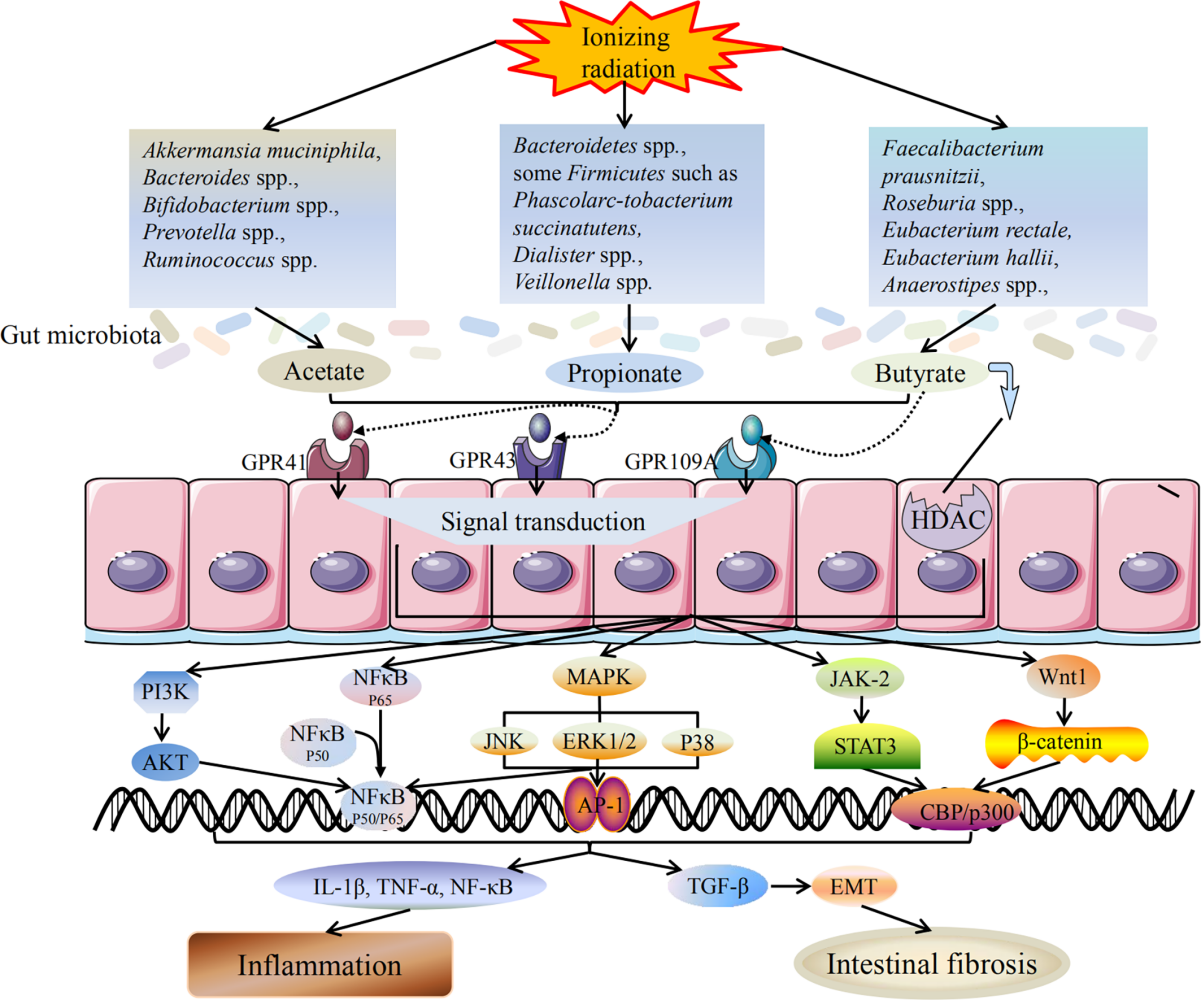
Mechanism of SCFAs on radiation-induced intestinal injury. Radiotherapy can lead to dysbiosis of the gut microbiota, including the changes in SCFAs-producing bacteria. The bacteria producing acetate, propionate and butyrate are all reduced, and the ability to produce SCFAs is weakened accordingly. SCFAs mainly induce downstream reactions through two pathways: activation of GPCRs and inhibition of HDAC. After activating GPCRs, SCFAs can act on downstream MAPK, NF-κB, PI3K/AKT, JAK/STAT, Wnt/β-catenin and other signaling pathways to promote signal transduction. SCFAs production is reduced and its anti-inflammatory activity is weakened, which in turn promotes the release of inflammatory cytokines and triggers inflammatory response. In addition, reduced production of SCFAs also induces TGF-β mediated intestinal fibrosis. Inflammation and intestinal fibrosis are two different outcomes of acute and chronic radiation-induced intestinal injury. Black arrows indicate promotion, and black lines indicate inhibition.

Intestinal fibrosis is the main intestinal complication in late radiotherapy. Kerem et al. (2006) have reported that oral dietary fiber or transrectal SCFAs can improve the healing of colon anastomosis and decrease the activity of matrix metalloproteinase-2 in rats after irradiation (Belizário et al., 2018). The results show that dietary pectin can significantly improve radiation-induced bowel fibrosis. The beneficial effects of SCFAs are manifold and involve at least two mechanisms, including activation of GPR41, GPR43, and GPR109A and inhibition of HDACs (Slavin, 2013; Chang et al., 2014). As an HDAC inhibitor, SCFAs can act as a regulator of gene expression by inducing protein hyperacetylation, chromatin remodeling, transcriptional activation and inhibition, leading to cell cycle arrest and cell death. Chung et al. have reported that SCFAs, as an HDAC inhibitor, can promote radiation-induced wound healing and improve skin fibrosis and tumorigenesis by inhibiting the expression of radiation-induced TGF-β and tumor TNF-α (Chung et al., 2004). Wang et al. have shown that sodium butyrate could attenuate TGF-β induced EMT and inhibit cell migration and invasion in hepatoma cells *in vitro*, and the effect of SCFAs on EMT and intestinal fibrosis may be partly attributed to its regulatory role as an HDAC inhibitor (Wang et al., 2013). Yang et al. have demonstrated that EMT plays a role in radiation-induced intestinal fibrosis and soluble dietary fiber may reduce radiation-induced EMT, and intestinal fibrosis by regulating intestinal flora and SCFA concentration (Yang et al., 2017). The mechanism of SCFAs on radiation-induced intestinal injury is shown in **Figure 1**.

## Therapeutic Options for Radiation-Induced Intestinal Injury

Gastrointestinal radiation injury is considered to be one of the important causes of systemic complications after radiation exposure, and may mediate some effects leading to multiple organ failure (François et al., 2013). In addition, in many experimental models, it has been shown that excessive inflammatory responses following intestine injury can also lead to multiple organ failure. Therefore, early intestinal changes that occur after radiation exposure are particularly promising targets for interventions to prevent or reduce radiation syndrome (Kim et al., 2009). Gut microbiota and its metabolites can be used as effective treatment options for radiation-induced intestinal injury. At present, the application of probiotics, fecal bacteria transplantation and metabolites play important roles in the protection of radiation-induced intestinal injury.

### Probiotics

There is sufficient research evidence that probiotics play an important role in preventing and treating cancer and complications. Many recent findings do support the hypothesis that daily use of certain selected probiotics can be an effective method to effectively protect patients from the risk of serious consequences caused by radiation therapy or chemotherapy. As a potential dietary supplement, probiotics may be able to reduce the risk of colorectal cancer and manage the safety of traditional cancer therapies such as surgery, radiation therapy, and chemotherapy (Drago, 2019; Shamekhi et al., 2020). Ciorba et al. showed that *Lactobacillu*s probiotics of sufficient dosage have the potential to reduce gastrointestinal toxicity after radiotherapy through clinical studies and preclinical models (Ciorba et al., 2015). Probiotics have been shown to play an important role in immune regulation and show anti-tumor properties. Bacterial strains may be responsible for the detection, and degradation of potential carcinogens and the production of SCFAs, which affect cell death and proliferation, and are called signaling molecules in the immune system. Lactic acid bacteria present in the intestine have been shown to play a role in the regression of carcinogenesis due to their influence on immune regulation, which can serve as evidence for the interaction between bacterial metabolites and immune epithelial cells. Probiotics have the ability to increase and decrease the production of anti-inflammatory cytokines, which play an important role in preventing cancer. They can also activate phagocytic cells to eliminate early cancer cells (Górska et al., 2019). *L.acidophilus* was also proved to be beneficial against the radiation-induced mucosal injury of intestine in rats (Ki et al., 2014). Moreover, probiotics supplements containing Bifidobacterium reduce chemotherapy-induced mucositis and radiation-induced diarrhea (Badgeley et al., 2021). The double strain probiotics (Lactobacillus acidophilus LAC-361 and Bifidobacterium longum BB-536) may reduce radiation induced diarrhea at the end of the treatment of patients with pelvic cancer (Demers et al., 2014). In a randomized trial, Garcia Peris and his colleagues (Garcia-Peris et al., 2016) showed that delivery of a fibrous mixture containing inulin promoted the growth of SCFA producing bacteria such as Roseburia and improved diarrhea in patients receiving pelvic radiotherapy (Kim et al., 2009). Therefore, it is of great significance that probiotics can mitigate radiation-induced intestinal injury.

### Fecal Microbiota Transplantation

Fecal microbiota transplantation (FMT) has been considered to affect microbial community due to radiation. Ding Xiao et al. have reported for the first time that it is safe and effective that FMT might improve intestinal symptoms and mucosal injury in patients with CRE for a period of time (Ding et al., 2020). FMT can improve the survival rate of irradiated animals, increase the peripheral white blood cell count and improve the gastrointestinal function and intestinal epithelial integrity of irradiated male and female mice. FMT retains the intestinal bacterial component of the host small intestine in a gender-specific manner, and retains its mRNA and long-term non-coding RNA expression profile. FMT can be used as a therapeutic agent to reduce the toxicity caused by radiation and improve the prognosis of tumor patients after radiotherapy (Cui et al., 2017). The post expansion of FMT in *Blautia* is beneficial to reduce intestinal inflammation and intestinal microbiome rebalancing (Jenq Robert et al., 2015; Wong et al., 2016). Although the relative proportion of these bacteria is low compared with other SCFA producers (such as *Faecalibacterium*), the trend of patients is that the production capacity of SCFA is higher but the dynamic decline, and the level of steady-state rectal mucosal cytokines involved in the maintenance of mucosal barrier is significantly reduced, which will be supported by the regulation of microbiota. Recently, FMT has been regarded as a successful treatment of immunotherapy induced colitis (Wang et al., 2018).

### Metabolites

#### SCFAs

Multiple evidence indicates that SCFAs are effective for the treatment of radiation-induced intestinal injury. Vernia et al. discovered early in clinical research that topical sodium butyrate enemas, at a dose of 80 mmol/L (80 mL/24 h), is effective for the treatment for acute radiation proctitis (Vernia et al., 2000). In the first controlled trial, Pinto A et al. provided safe and effective evidence that SCFA enemas contained 60 mM sodium acetate, 30 mM sodium propionate, and 40 mM sodium butyrate, which can accelerate healing process of chronic radiation proctitis (Pinto et al., 1999). Later, it was confirmed by research that patients with chronic radiation rectal injury were given SCFAs enema, and the clinical symptoms of radiation rectal disease were significantly relieved (Hong et al., 2001). Clinical studies showed that daily prophylactic use of sodium butyrate enemas did not reduce the incidence, severity and duration of acute radiation proctitis (Maggio et al., 2014). Among SCFAs, valeric acid plays the most important role in radioprotection. In detail, exogenous valeric acid supplementation improves the survival rate of irradiated mice, protects hematopoietic organs, and improves gastrointestinal function and intestinal epithelial integrity of irradiated mice. High-throughput sequencing and relative and absolute quantitative isobaric tags indicated that oral valeric acid restored the taxonomic ratio of enteric bacteria, and reprogrammed the small intestine protein profile of mice exposed to systemic irradiation. Valeric acid has a beneficial effect on radiation-induced hematopoiesis and intestinal damage in which it reduces inflammation and protects the intestinal bacterial composition of irradiated animals. Importantly, keratin 1 (KRT1) plays a key role in the radioprotection of valeric acid *in vivo* and *in vitro*. Valeric acid may play a role in AML1/KRT1 signaling through transporters or CPCRs (Li et al., 2020). Moreover, as one of the SCFAs, valproic acid has been found to have anti-tumor activity, the main mechanism of which is to inhibit histone deacetylase (Gurvich et al., 2004). Debeb et al. have reported that valproic acid can be used as a radiosensitizer for breast cancer in the short term, but it may increase the risk of cancer recurrence in the long term (Debeb et al., 2010). Chen et al. have also found that low dose valproic acid enhances radiosensitivity of prostate cancer by acetylating p53 dependent mitochondrial membrane potential and apoptosis (Chen et al., 2011).

#### Other Metabolites

Omega-3 (n-3) polyunsaturated fatty acids (PUFA) can reverse intestinal microbial disease by increasing the number of beneficial bacterial species including *Lactobacilli*, *Bifidobacteria*, and butyrate-producing bacteria such as *Roseburia* and *Coprococcus*. In addition, n-3 PUFA reduces the proportion of LPS production and mucolytic bacteria in the intestine, which can reduce inflammation and oxidative stress. Importantly, n-3 PUFA also plays an anti-cancer role in colorectal cancer (Zhang et al., 2019). Lee et al. have found that oral consumption of human symbiotic bacteria containing lactic acid-producing bacteria, such as *Bifidobacterium* and *Lactobacillus*, can significantly promote the proliferation of Lgr5^+^ intestinal stem cells and epithelial cells *in vivo* and *in vitro*. Among several metabolites of lactic acid producing bacteria symbionts, lactic acid is related to epithelial development mediated by intestinal stem cells. Paneth cells and intestinal stromal cells highly express Gpr81 (a known lactate receptor), and lactic acid treatment promotes intestinal stem cell-mediated epithelial regeneration in a Gpr81-dependent manner. In addition, feeding lactic acid-producing bacterial symbionts or lactic acid can protect mice from severe intestinal damage caused by radiation exposure and chemotherapy treatment. Similarly, they also confirmed that lactate and the receptor Gpr81 stimulate the Wnt/β-catenin signaling pathway in Paneth and intestinal stromal cells to promote intestinal stem cell-mediated epithelial regeneration (Lee et al., 2018).

## Conclusions and Perspectives

**Figure 2:**
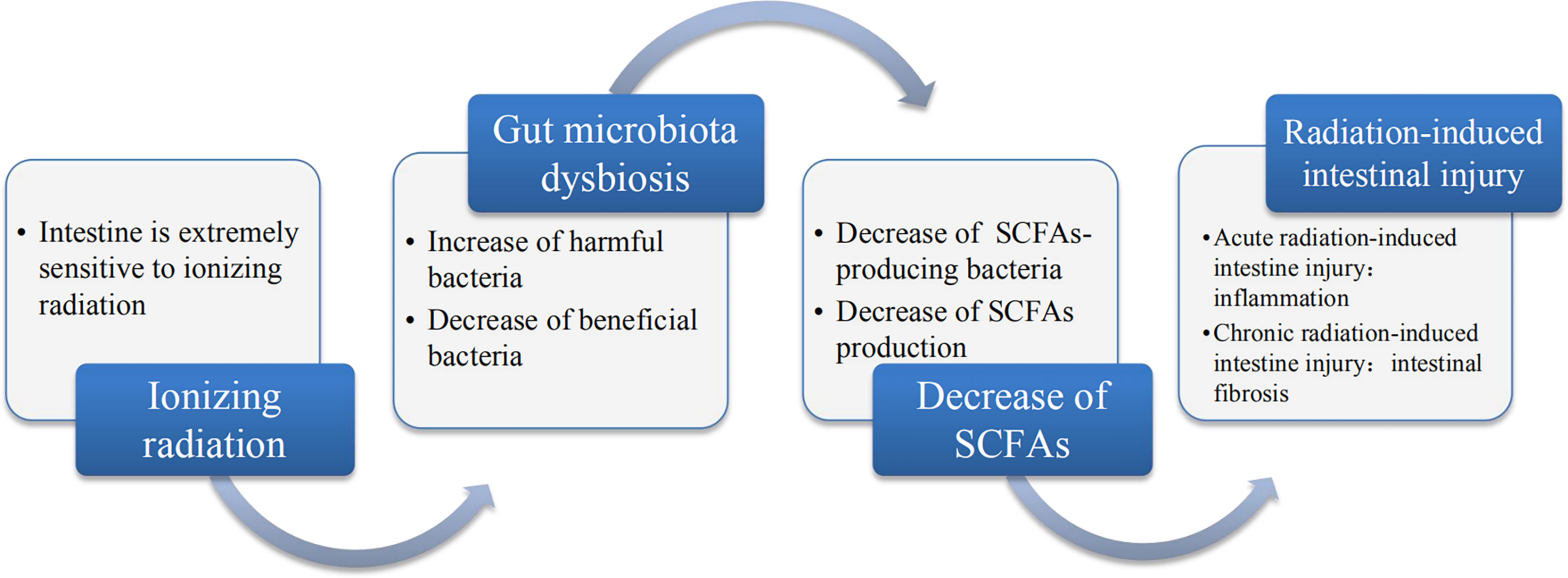
The relationship between ionizing radiation, gut microbiota, SCFAs and radiation-induced intestinal injury. Ionizing radiation can lead to dysbiosis of gut microbiota, which is mainly disruption and it leads to the decrease of beneficial bacteria and increase in harmful or pathogenic bacteria. Gut microbiota dysbiosis includes the decrease in SCFAs-producing bacteria, which may result in the decrease of SCFAs. The decrease of SCFAs may be a major pathogenesis of radiation-induced intestinal injury.

An increasing number of evidence demonstrated that radiotherapy can lead to gut microbiota dysbiosis and cause the alterations in the gut microbial communities such as the alterations of *Bacteroidetes* and *Firmicutes* counts, which disrupts intestinal homeostasis and thereby promoting the occurrence and development of various diseases. Therefore, it has attracted more and more attention to reveal the pathogenesis of the disease from the perspective of gut microbiota. In addition, due to the different radiation sensitivity of different species, the alterations in gut microbiota caused by ionizing radiation are also different. Most importantly, the gut microbiota metabolites such as SCFAs play a pivotal role in health and disease. Radiation-induced gut microbiota dysbiosis causes changes in SCFAs accordingly and the reason can be attributed to the fact that radiotherapy has led to changes in the SCFAs-producing bacteria such as *Bacteroidetes*, *Firmicutes* and *Roseburia* etc. which in turn led to a decrease in the production of SCFAs. Furthermore, many studies have confirmed the correlation between the alterations of SCFAs and radiation-induced intestinal injury. The crosstalk between the alterations of SCFAs and radiation-induced intestinal injury can be traced from upstream to the decrease of the diversity of gut microbiota that produces SCFAs, and the downstream SCFAs can affect the occurrence and development of radiation-induced intestinal injury through different mechanisms. In this review, we focused on the progressive relationship among ionizing radiation, gut microbiota, SCFAs and radiation-induced intestinal injury, as shown in **Figure 2**. Furthermore, we revealed the mechanism of radiation-induced intestinal injury from the perspective of gut microbiota and its metabolite SCFAs, providing a reference for clinical diagnosis and treatment in the future. Of course, probiotics, FMT and various metabolites also provide good prospects for the treatment of radiation-induced intestinal injury, which further proves the importance of gut microbiota in maintaining intestinal homeostasis and human health.

In conclusion, we believe that radiotherapy can cause changes in the gut microbiota, including the SCFAs-producing bacteria, which in turn leads to a decrease in SCFAs production. Less production of SCFAs is likely to be the pathogenic mechanism of radiation-induced intestinal injury and supplementing SCFAs can alleviate radiation intestinal injury, which can provide a new reference for the pathogenesis and treatment of radiation-induced intestine injury. In short, in this review, we highlight the correlation among gut microbiota, SCFAs and radiation-induced intestinal injury exposed to ionizing radiation. We also elaborate this relevance and have a better understanding of the pathophysiological basis of gut microbiota and SCFAs associated with radiation-induced intestinal injury so as to provide a reference for accurate treatment in the future.

## Author Contributions

YQL and LYZ provide guidance on ideas and directions as well as the revision of the article. JPH and ND are responsible for the guidance of methodology. YYL and YMZ are responsible for the writing of the article. Others are responsible for collecting materials and participating in the revision of the article. All authors contributed to the article and approved the submitted version.

## Funding

This project was supported by the National Natural Science Foundation of China (grant numbers 81973595, 82004094), China Postdoctoral Science Foundation Project (grant numbers 2021M693794), The Open Fund Project of Key Laboratory of Dunhuang Medicine and Translational Education Ministry (grant numbers DHYX19-13).

## Conflict of Interest

The authors declare that the research was conducted in the absence of any commercial or financial relationships that could be construed as a potential conflict of interest.

## References

[B1] Afrin S. Giampieri F. Gasparrini M. Forbes–Hernández T. Y. Cianciosi D. Reboredo–Rodriguez P.. (2020). Dietary Phytochemicals in Colorectal Cancer Prevention and Treatment: A Focus on the Molecular Mechanisms Involved. Biotechnol. Adv. 38, 107322. 10.1016/j.biotechadv.2018.11.011 30476540

[B2] Badgeley A. Anwar H. Modi K. Murphy P. Lakshmikuttyamma A. (2021). Effect of Probiotics and Gut Microbiota on Anti-Cancer Drugs: Mechanistic Perspectives. Biochim. Biophys. Acta Rev. Cancer 1875 (1), 188494. 10.1016/j.bbcan.2020.188494 33346129

[B3] Belizário J. E. Faintuch J. Garay-Malpartida M. (2018). Gut Microbiome Dysbiosis and Immunometabolism: New Frontiers for Treatment of Metabolic Diseases. Mediators Inflammation 2018, 2037838. 10.1155/2018/2037838 PMC6304917 30622429

[B4] Bose S. Ramesh V. Locasale J. W. (2019). Acetate Metabolism in Physiology, Cancer, and Beyond. Trends Cell Biol. 29 (9), 695–703. 10.1016/j.tcb.2019.05.005 31160120 PMC6699882

[B5] Bouter K. E. van Raalte Daniël H. Groen A. K. Nieuwdorp M. (2017). Role of the Gut Microbiome in the Pathogenesis of Obesity and Obesity-Related Metabolic Dysfunction. Gastroenterology 152, 1671–1678. 10.1053/j.gastro.2016.12.048 28192102

[B6] Budden K. F. Gellatly S. L. Wood D. L. A. Cooper M. A. Morrison M. Hugenholtz P.. (2017). Emerging Pathogenic Links Between Microbiota and the Gut-Lung Axis. Nat. Rev. Microbiol. 15, 55–63. 10.1038/nrmicro.2016.142 27694885

[B7] Carbonero F. Mayta-Apaza A. C. Yu J. Z. Lindeblad M. Lyubimov A. Neri F.. (2018). A Comparative Analysis of Gut Microbiota Disturbances in the Gottingen Minipig and Rhesus Macaque Models of Acute Radiation Syndrome Following Bioequivalent Radiation Exposures. Radiat. Environ. Biophys. 57 (4), 419–426. 10.1007/s00411-018-0759-0 30343431

[B8] Carbonero F. Mayta A. Bolea M. Yu J. Z. Lindeblad M. Lyubimov A.. (2019). Specific Members of the Gut Microbiota are Reliable Biomarkers of Irradiation Intensity and Lethality in Large Animal Models of Human Health. Radiat. Res. 191 (1), 107–121. 10.1667/RR14975.1 30430918

[B9] Casero D. Gill K. Sridharan V. Koturbash I. Nelson G. Hauer–Jensen M.. (2017). Space-Type Radiation Induces Multimodal Responses in the Mouse Gut Microbiome and Metabolome. Microbiome 5 (1), 105. 10.1186/s40168-017-0325-z 28821301 PMC5563039

[B10] Castaño-Rodríguez N. Underwood A. P. Merif J. Riordan S. M. Rawlinson W. D. Mitchell H. M.. (2018). Gut Microbiome Analysis Identifies Potential Etiological Factors in Acute Gastroenteritis. Infect. Immun. 86 (7), e00060–e00018. 10.1128/IAI.00060-18 29685983 PMC6013661

[B11] Chang P. V. Hao L. Offermanns S. Medzhitov R. (2014). The Microbial Metabolite Butyrate Regulates Intestinal Macrophage Function *via* Histone Deacetylase Inhibition. Proc. Natl. Acad. Sci. U. S. A. 111 (6), 2247–2252. 10.1073/pnas.1322269111 24390544 PMC3926023

[B12] Chassaing B. Cascales E. (2018). Antibacterial Weapons: Targeted Destruction in the Microbiota. Trends Microbiol. 26 (4), 329–338. 10.1016/j.tim.2018.01.006 29452951

[B13] Chater C. Saudemont A. Zerbib P. (2019). Chronic Radiation Enteritis. J. Visc. Surg. 156 (2), 175–176. 10.1016/j.jviscsurg.2018.09.002 30249429

[B14] Chen X. Wong J. Y. Wong P. Radany E. H. (2011). Low-Dose Valproic Acid Enhances Radiosensitivity of Prostate Cancer Through Acetylated P53-Dependent Modulation of Mitochondrial Membrane Potential and Apoptosis. Mol. Cancer Res. 9 (4), 448–461. 10.1158/1541-7786.MCR-10-0471 21303901 PMC3655769

[B15] Chen J. Zhao K. N. Vitetta L. (2019). Effects of Intestinal Microbial-Elaborated Butyrate on Oncogenic Signaling Pathways. Nutrients 11 (5), 1026. 10.3390/nu11051026 PMC6566851 31067776

[B16] Chung Y. L. Wang A. J. Yao L. F. (2004). Antitumor Histone Deacetylase Inhibitors Suppress Cutaneous Radiation Syndrome: Implications for Increasing Therapeutic Gain in Cancer Radiotherapy. Mol. Cancer Ther. 3 (3), 317–325. 15026552

[B17] Ciorba M. A. Hallemeier C. L. Stenson W. F. Parikh P. J. (2015). Probiotics to Prevent Gastrointestinal Toxicity From Cancer Therapy: An Interpretive Review and Call to Action. Curr. Opin. Support Palliat Care 9 (2), 157–162. 10.1097/SPC.0000000000000134 25872116 PMC4852157

[B18] Citrin D. E. (2017). Recent Developments in Radiotherapy. N. Engl. J. Med. 377 (11), 1065–1075. 10.1056/NEJMra1608986 28902591

[B19] Cook S. I. Sellin J. H. (1998). Review Article: Short Chain Fatty Acids in Health and Disease. Aliment Pharmacol. Ther. 12 (6), 499–507. 10.1046/j.1365-2036.1998.00337.x 9678808

[B20] Crawford P. A. Gordon J. I. (2005). Microbial Regulation of Intestinal Radiosensitivity. Proc. Natl. Acad. Sci. U.S.A. 102 (37), 13254–13259. 10.1073/pnas.0504830102 16129828 PMC1193536

[B21] Cui M. Xiao H. Li Y. Zhou L. Zhao S. Luo D.. (2017). Faecal Microbiota Transplantation Protects Against Radiation-Induced Toxicity. EMBO Mol. Med. 9 (4), 448–461. 10.15252/emmm.201606932 28242755 PMC5376756

[B22] Debeb B. G. Xu W. Mok H. Li L. Robertson F. Ueno N. T.. (2010). Differential Radiosensitizing Effect of Valproic Acid in Differentiation Versus Self-Renewal Promoting Culture Conditions. Int. J. Radiat. Oncol. Biol. Phys. 76 (3), 889–895. 10.1016/j.ijrobp.2009.09.052 20159363 PMC2892870

[B23] De Filippo C. Cavalieri D. Di Paola M. Ramazzotti M. Poullet J. B. Massart S.. (2010). Impact of Diet in Shaping Gut Microbiota Revealed by a Comparative Study in Children From Europe and Rural Africa. Proc. Natl. Acad. Sci. U.S.A. 107 (33), 14691–14696. 10.1073/pnas.1005963107 20679230 PMC2930426

[B24] Demers M. Dagnault A. Desjardins J. (2014). A Randomized Double-Blind Controlled Trial: Impact of Probiotics on Diarrhea in Patients Treated With Pelvic Radiation. Clin. Nutr. 33 (5), 761–767. 10.1016/j.clnu.2013.10.015 24200199

[B25] Denton A. Forbes A. Andreyev J. Maher E. J. (2002). Non Surgical Interventions for Late Radiation Proctitis in Patients Who Have Received Radical Radiotherapy to the Pelvis. Cochrane Database Syst. Rev. (1), CD003455. 10.1002/14651858.CD003455 11869662

[B26] De Ruysscher D. Niedermann G. Burnet N. G. Siva S. Lee A. W. M. Hegi-Johnson F. (2019). Radiotherapy Toxicity. Nat. Rev. Dis. Primers 5 (1), 13. 10.1038/s41572-019-0064-5 30792503

[B27] Ding X. Li Q. Li P. Chen X. Xiang L. Bi L.. (2020). Fecal Microbiota Transplantation: A Promising Treatment for Radiation Enteritis? Radiother. Oncol. 143, 12–18. 10.1016/j.radonc.2020.01.011 32044171

[B28] Dostal A. Chassard C. Hilty F. M. Zimmermann M. Jaeggi B. Rossi T. S.. (2012). Iron Depletion and Repletion With Ferrous Sulfate or Electrolytic Iron Modifies the Composition and Metabolic Activity of the Gut Microbiota in Rats. J. Nutr. 142, 271–277. 10.3945/jn.111.148643 22190022 PMC3260059

[B29] Drago L. (2019). Probiotics and Colon Cancer. Microorganisms 7 (3), 66. 10.3390/microorganisms7030066 PMC6463067 30823471

[B30] Duncan S. H. Barcenilla A. Stewart C. S. Pryde S. E. Flint H. J. (2002). Acetate Utilization and Butyryl Coenzyme A (CoA):acetate-CoA Transferase in Butyrate-Producing Bacteria From the Human Large Intestine. Appl. Environ. Microbiol. 68 (10), 5186–5190. 10.1128/AEM.68.10.5186-5190.2002 12324374 PMC126392

[B31] Feng W. Ao H. Peng C. (2018). Gut Microbiota, Short-Chain Fatty Acids, and Herbal Medicines. Front. Pharmacol. 9, 1354. 10.3389/fphar.2018.01354 30532706 PMC6265305

[B32] Feng Q. Chen W. D. Wang Y. D. (2018). Gut Microbiota: An Integral Moderator in Health and Disease. Front. Microbiol. 9, 151. 10.3389/fmicb.2018.00151 29515527 PMC5826318

[B33] Ferreira M. R. Muls A. Dearnaley D. P. Andreyev H. J. (2014). Microbiota and Radiation-Induced Bowel Toxicity: Lessons From Inflammatory Bowel Disease for the Radiation Oncologist. Lancet Oncol. 15 (3), e139–e147. 10.1016/S1470-2045(13)70504-7 24599929

[B34] Ferrer-Mayorga G. Larriba M. J. Crespo P. Muñoz A. (2019). Mechanisms of Action of Vitamin D in Colon Cancer. J. Steroid Biochem. Mol. Biol. 185, 1–6. 10.1016/j.jsbmb.2018.07.002 29981368

[B35] François A. Milliat F. Guipaud O. Benderitter M. (2013). Inflammation and Immunity in Radiation Damage to the Gut Mucosa. BioMed. Res. Int. 2013, 123241. 10.1155/2013/123241 23586015 PMC3614034

[B36] Garcia-Peris P. Velasco C. Hernandez M. Lozano M. A Paron L. de la Cuerda C.. (2016). Effect of Inulin and Fructo-Oligosaccharide on the Prevention of Acute Radiation Enteritis in Patients With Gynecological Cancer and Impact on Quality-of-Life: A Randomized, Double-Blind, Placebo-Controlled Trial. Eur. J. Clin. Nutr. 70 (2), 170–174. 10.1038/ejcn.2015.192 26603881

[B37] Gecse Krisztina B. (2018). Vermeire Severine,Differential Diagnosis of Inflammatory Bowel Disease: Imitations and Complications. J. Lancet. Gastroenterol. Hepatol. 3, 644–653. 10.1016/S2468-1253(18)30159-6 30102183

[B38] Gentile C. L. Weir T. L. (2018). The Gut Microbiota at the Intersection of Diet and Human Health. Science 362 (6416), 776–780. 10.1126/science.aau5812 30442802 PMC13264711

[B39] Gerassy-Vainberg S. Blatt A. Danin-Poleg Y. Gershovich K. Sabo E. Nevelsky A.. (2018). Radiation Induces Proinflammat Ory Dysbiosis: Transmission of Inflammatory Susceptibility by Host Cytokine Induction. J. Gut 67, 97–107. 10.1136/gutjnl-2017-313789 28438965

[B40] Górska A. Przystupski D. Niemczura M. J. Kulbacka J. (2019). Probiotic Bacteria: A Promising Tool in Cancer Prevention and Therapy. Curr. Microbiol. 76 (8), 939–949. 10.1007/s00284-019-01679-8 30949803 PMC6586914

[B41] Goudarzi M. Mak T. D. Jacobs J. P. Moon B. H. Strawn S. J. Braun J.. (2016). An Integrated Multi-Omic Approach to Assess Radiation Injury on the Host-Microbiome Axis. Radiat. Res. 186 (3), 219–234. 10.1667/RR14306.1 27512828 PMC5304359

[B42] Guo H. Chou W. C. Lai Y. Liang K. Tam J. W. Brickey W. J.. (2020). Multi-Omics Analyses of Radiation Survivors Identify Radioprotective Microbes and Metabolites. Science 370 (6516), eaay9097. 10.1126/science.aay9097 33122357 PMC7898465

[B43] Gurvich N. Tsygankova O. M. Meinkoth J. L. Klein P. S. (2004). Histone Deacetylase is a Target of Valproic Acid-Mediated Cellular Differentiation. Cancer Res. 64 (3), 1079–1086. 10.1158/0008-5472.CAN-03-0799 14871841

[B44] Hauer-Jensen M. Denham J. W. Andreyev H. J. (2014). Radiation Enteropathy–Pathogenesis, Treatment and Prevention. Nat. Rev. Gastroenterol. Hepatol. 11 (8), 470–479. 10.1038/nrgastro.2014.46 24686268 PMC4346191

[B45] Haydont V. Vozenin-Brotons M. C. (2007). Maintenance of Radiation-Induced Intestinal Fibrosis: Cellular and Molecular Features. World J. Gastroenterol. 13 (19), 2675–2683. 10.3748/wjg.v13.i19.2675 17569135 PMC4147115

[B46] Hetzel M. Brock M. Selmer T. Pierik A. J. Golding B. T. Buckel W. (2003). Acryloyl-CoA Reductase From Clostridium Propionicum. An Enzyme Complex of Propionyl-CoA Dehydrogenase and Electron-Transferring Flavoprotein. Eur. J. Biochem. 270 (5), 902–910. 10.1046/j.1432-1033.2003.03450.x 12603323

[B47] Hong J. J. Park W. Ehrenpreis E. D. (2001). Review Article: Current Therapeutic Options for Radiation Proctopathy. Aliment Pharmacol. Ther. 15 (9), 1253–1262. 10.1046/j.1365-2036.2001.01075.x 11552895

[B48] Hou B. Xu Z. W. Zhang C. G. (2007). The Effects of Gut Commensal Bacteria Depletion on Mice Exposed to Acute Lethal Irradiation. J. Radiat. Res. 48 (4), 347–350. 10.1269/jrr.07020 17598956

[B49] Husebye E. Skar V. Høverstad T. Iversen T. Melby K. (1995). Abnormal Intestinal Motor Patterns Explain Enteric Colonization With Gram-Negative Bacilli in Late Radiation Enteropathy. Gastroenterology 109 (4), 1078–1089. 10.1016/0016-5085(95)90565-0 7557072

[B50] Husted A. S. Trauelsen M. Rudenko O. Hjorth S. A. Schwartz T. W. (2017). GPCR-Mediated Signaling of Metabolites. Cell Metab. 25, 777–796. 10.1016/j.cmet.2017.03.008 28380372

[B51] Jenq Robert R. Taur Y. Devlin Sean M. Ponce D. M. Goldberg J. D. Ahr K. F.. (2015). Intestinal Blautia Is Associated With Reduced Death From Graft-Versus-Host Disease. Biol. Blood Marrow Transplant. 21, 1373–1383. 10.1016/j.bbmt.2015.04.016 25977230 PMC4516127

[B52] Karki R. Malireddi R. K. S. Zhu Q. Kanneganti T. D. (2017). NLRC3 Regulates Cellular Proliferation and Apoptosis to Attenuate the Development of Colorectal Cancer. Cell Cycle 16 (13), 1243–1251. 10.1080/15384101.2017.1317414 28598238 PMC5531621

[B53] Kerem M. Bedirli A. Karahacioglu E. Pasaoglu H. Sahin O. Bayraktar N.. (2006). Effects of Soluble Fiber on Matrix Metalloproteinase-2 Activity and Healing of Colon Anastomosis in Rats Given Radiotherapy. Clin. Nutr. 25 (4), 661–670. 10.1016/j.clnu.2006.01.028 16677740

[B54] Kiang J. G. Olabisi A. O. (2019). Radiation: A Poly-Traumatic Hit Leading to Multi-Organ Injury. Cell Biosci. 9, 25. 10.1186/s13578-019-0286-y 30911370 PMC6417034

[B55] Ki Y. Kim W. Cho H. Ahn K. Choi Y. Kim D. (2014). The Effect of Probiotics for Preventing Radiation-Induced Morphological Changes in Intestinal Mucosa of Rats. J. Korean Med. Sci. 29 (10), 1372–1378. 10.3346/jkms.2014.29.10.1372 25368490 PMC4214937

[B56] Kim Y. S. Kim J. Park S. J. (2015). High-Throughput 16S rRNA Gene Sequencing Reveals Alterations of Mouse Intestinal Microbiota After Radiotherapy. Anaerobe 33, 1–7. 10.1016/j.anaerobe.2015.01.004 25600706

[B57] Kim S. H. Lee H. J. Kim J. S. Moon C. Kim J. C. Park H. R.. (2009). Protective Effect of an Herbal Preparation (HemoHIM) on Radiation-Induced Intestinal Injury in Mice. J. Med. Food. 12 (6), 1353–1358. 10.1089/jmf.2008.1322 20041793

[B58] Knauf F. Brewer J. R. Flavell R. A. (2019). Immunity, Microbiota and Kidney Disease. Nat. Rev. Nephrol. 15 (5), 263–274. 10.1038/s41581-019-0118-7 30796361

[B59] Koh A. De Vadder F. Kovatcheva-Datchary P. Bäckhed F. (2016). From Dietary Fiber to Host Physiology: Short-Chain Fatty Acids as Key Bacterial Metabolites. Cell 165 (6), 1332–1345. 10.1016/j.cell.2016.05.041 27259147

[B60] Kumagai T. Rahman F. Smith A. M. (2018). The Microbiome and Radiation Induced-Bowel Injury: Evidence for Potential Mechanistic Role in Disease Pathogenesis. Nutrients 10 (10), 1405. 10.3390/nu10101405 PMC6213333 30279338

[B61] Lam V. Moulder J. E. Salzman N. H. Dubinsky E. A. Andersen G. L. Baker J. E. (2012). Intestinal Microbiota as Novel Biomarkers of Prior Radiation Exposure. Radiat. Res. 177 (5), 573–583. 10.1667/RR2691.1 22439602

[B62] Lavrinienko A. Mappes T. Tukalenko E. Mousseau E. Møller E. Knight R.. (2018). Environmental Radiation Alters the Gut Microbiome of the Bank Vole Myodes Glareolus. ISME J. 12 (11), 2801–2806. 10.1038/s41396-018-0214-x 29988064 PMC6193954

[B63] Lee Y. S. Kim T. Y. Kim Y. Lee S. H. Kim S. Kang S. W.. (2018). Microbiota-Derived Lactate Accelerates Intestinal Stem-Cell-Mediated Epithelial Development. Cell Host Microbe 24 (6), 833–846.e6. 10.1016/j.chom.2018.11.002 30543778

[B64] Ley R. E. Turnbaugh P. J. Klein S. Gordon J. I. (2006). Microbial Ecology: Human Gut Microbes Associated With Obesity. Nature 444 (7122), 1022–1023. 10.1038/4441022a 17183309

[B65] Li Y. Dong J. Xiao H. Zhang S. Wang B. Cui M.. (2020). Gut Commensal Derived-Valeric Acid Protects Against Radiation Injuries [Published Online Ahead of Print, 2020 Jan 13]. Gut Microbes, 1–18. 10.1080/19490976.2019.1709387 PMC7524389 31931652

[B66] Litvak Y. Byndloss Mariana X. Tsolis R. M. Bäumler A. J. (2017). Dysbiotic Proteobacteria Expansion: A Microbial Signature of Epithelial Dysfunction. Curr. Opin. Microbiol. 39, 1–6. 10.1016/j.mib.2017.07.003 28783509

[B67] Liu H. Wang J. He T. Becker S. Zhang G. Li D.. (2018). Butyrate: A Double-Edged Sword for Health? Adv. Nutr. 9 (1), 21–29. 10.1093/advances/nmx009 29438462 PMC6333934

[B68] Liu X. Zhou Y. Wang S. Guan H. Hu S. Huang R.. (2019). Impact of Low-Dose Ionising Radiation on the Composition of the Gut Microbiota of Mice. Toxicol. Sci., kfz144. 10.1093/toxsci/kfz144 31236581

[B69] Louis P. Hold G. L. Flint H. J. (2014). The Gut Microbiota, Bacterial Metabolites and Colorectal Cancer. Nat. Rev. Microbiol. 12 (10), 661–672. 10.1038/nrmicro3344 25198138

[B70] Lupp C. Robertson M. L. Wickham M. E. Sekirov I. Champion O. L. Gaynor E. C.. (2007). Host-Mediated Inflammation Disrupts the Intestinal Microbiota and Promotes the Overgrowth of Enterobacteriaceae. Cell Host Microbe. 2(2), 119–129. 10.1016/j.chom.2007.06.010 18005726

[B71] Lynch Susan V. Pedersen O. (2016). The Human Intestinal Microbiome in Health and Disease. J. N. Engl. J. Med. 375, 2369–2379. 10.1056/NEJMra1600266 27974040

[B72] Maggio A. Magli A. Rancati T. Fiorino C. Valvo F. Fellin G.. (2014). Daily Sodium Butyrate Enema for the Prevention of Radiation Proctitis in Prostate Cancer Patients Undergoing Radical Radiation Therapy: Results of a Multicenter Randomized Placebo-Controlled Dose-Finding Phase 2 Study. Int. J. Radiat. Oncol. Biol. Phys. 89 (3), 518–524. 10.1016/j.ijrobp.2014.03.018 24803033

[B73] Mangoni M. Sottili M. Gerini C. Desideri I. Bastida C. Pallotta S.. (2017). A PPAR-Gamma Agonist Protects From Radiation-Induced Intestinal Toxicity. United Eur. Gastroenterol. J. 5 (2), 218–226. 10.1177/2050640616640443 PMC5349355 28344789

[B74] Manichanh C. Varela E. Martinez C. Antolin M. Llopis M. Doré J.. (2008). The Gut Microbiota Predispose to the Pathophysiology of Acute Postradiotherapy Diarrhea. Am. J. Gastroenterol. 103 (7), 1754–1761. 10.1111/j.1572-0241.2008.01868.x 18564125

[B75] Marchesi J. R. Adams D. H. Fava F. Hermes G. D. Hirschfield G. M. Hold G.. (2016). The Gut Microbiota and Host Health: A New Clinical Frontier. Gut 65 (2), 330–339. 10.1136/gutjnl-2015-309990 26338727 PMC4752653

[B76] Mármol I. Sánchez-de-Diego C. Pradilla Dieste A. Cerrada E. Rodriguez Yoldi M. J. (2017). Colorectal Carcinoma: A General Overview and Future Perspectives in Colorectal Cancer. Int. J. Mol. Sci. 18 (1), 197. 10.3390/ijms18010197 PMC5297828 28106826

[B77] McOrist A. L. Miller R. B. Bird A. R. Keogh J. B. Noakes M. Topping D. L.. (2011). Fecal Butyrate Levels Vary Widely Among Individuals But are Usually Increased by a Diet High in Resistant Starch. J. Nutr. 141 (5), 883–889. 10.3945/jn.110.128504 21430242

[B78] Miller T. L. Wolin M. J. (1996). Pathways of Acetate, Propionate, and Butyrate Formation by the Human Fecal Microbial Flora. Appl. Environ. Microbiol. 62 (5), 1589–1592. 10.1128/aem.62.5.1589-1592.1996 8633856 PMC167932

[B79] Morgan J. L. Ritchie L. E. Crucian B. E. Theriot C. Wu H. Sams C.. (2014). Increased Dietary Iron and Radiation in Rats Promote Oxidative Stress, Induce Localized and Systemic Immune System Responses, and Alter Colon Mucosal Environment. FASEB J. 28 (3), 1486–1498. 10.1096/fj.13-239418 24334706

[B80] Mosca A. Leclerc M. Hugot J. P. (2016). Gut Microbiota Diversity and Human Diseases: Should We Reintroduce Key Predators in Our Ecosystem? Front. Microbiol. 7, 455. 10.3389/fmicb.2016.00455 27065999 PMC4815357

[B81] Nam Y. D. Kim H. J. Seo J. G. Kang S. W. Bae J. W. (2013). Impact of Pelvic Radiotherapy on Gut Microbiota of Gynecological Cancer Patients Revealed by Massive Pyrosequencing. PloS One 8 (12), e82659. 10.1371/journal.pone.0082659 24367534 PMC3867375

[B82] Park M. Kwon J. Shin H. J. Moon S. M. Kim S. B. Shin U. S.. (2020). Butyrate Enhances the Efficacy of Radiotherapy *via* FOXO3A in Colorectal Cancer Patient−Derived Organoids. Int. J. Oncol. 57 (6), 1307–1318. 10.3892/ijo.2020.5132 33173975 PMC7646587

[B83] Perona M. Thomasz L. Rossich L. Rodriguez C. Pisarev M. A. Rosemblit C.. (2018). Radiosensitivity Enhancement of Human Thyroid Carcinoma Cells by the Inhibitors of Histone Deacetylase Sodium Butyrate and Valproic Acid. Mol. Cell Endocrinol. 478, 141–150. 10.1016/j.mce.2018.08.007 30125607

[B84] Pía de la Maza M. Gotteland M. Ramírez C. Araya M. Yudin T. Bunout D.. (2001). Acute Nutritional and Intestinal Changes After Pelvic Radiation. J. Am. Coll. Nutr. 20 (6), 637–642. 10.1080/07315724.2001.10719161 11771680

[B85] Pinto A. Fidalgo P. Cravo M. Midões J. Chaves P. Rosa J.. (1999). Short Chain Fatty Acids are Effective in Short-Term Treatment of Chronic Radiation Proctitis: Randomized, Double-Blind, Controlled Trial. Dis. Colon Rectum. 42 (6), 788–796. 10.1007/BF02236937 10378604

[B86] Postler T. S. Ghosh S. (2017). Understanding the Holobiont: How Microbial Metabolites Affect Human Health and Shape the Immune System. Cell Metab. 26 (1), 110–130. 10.1016/j.cmet.2017.05.008 28625867 PMC5535818

[B87] Ragsdale S. W. Pierce E. (2008). Acetogenesis and the Wood-Ljungdahl Pathway of CO(2) Fixation. Biochim. Biophys. Acta 1784 (12), 1873–1898. 10.1016/j.bbapap.2008.08.012 18801467 PMC2646786

[B88] Ratajczak W. Rył A. Mizerski A. Walczakiewicz K. Sipak O. Laszczyńska M. (2019). Immunomodulatory Potential of Gut Microbiome-Derived Short-Chain Fatty Acids (SCFAs). Acta Biochim. Pol. 66 (1), 1–12. 10.18388/abp.2018_2648 30831575

[B89] Reichardt N. Duncan S. H. Young P. Belenguer A. McWilliam Leitch C. Scott K. P.. (2014). Phylogenetic Distribution of Three Pathways for Propionate Production Within the Human Gut Microbiota. ISME J. 8 (6), 1323–1335. 10.1038/ismej.2014.14 24553467 PMC4030238

[B90] Reis Ferreira M. Andreyev H. J. N. Mohammed K. Truelove L. Gowan S. M. Li J.. (2019). Microbiota- and Radiotherapy-Induced Gastrointestinal Side-Effects (MARS) Study: A Large Pilot Study of the Microbiome in Acute and Late-Radiation Enteropathy. Clin. Cancer Res. 25 (21), 6487–6500. 10.1158/1078-0432.CCR-19-0960 31345839

[B91] Rosoff C. B. (1963). The Role of Intestinal Bacteria in the Recovery From Whole Body Radiation. J. Exp. Med. 118 (6), 935–943. 10.1084/jem.118.6.935 14112272 PMC2137691

[B92] Salonen A. Lahti L. Salojärvi J. Holtrop G. Korpela K. Duncan S. H.. (2014). Impact of Diet and Individual Variation on Intestinal Microbiota Composition and Fermentation Products in Obese Men. ISME J. 8 (11), 2218–2230. 10.1038/ismej.2014.63 24763370 PMC4992075

[B93] Segain J. P. Raingeard de la Blétière D. Bourreille A. Leray V. Gervois N. Rosales C.. (2000). Butyrate Inhibits Inflammatory Responses Through NFkappaB Inhibition: Implications for Crohn’s Disease. Gut. 47 (3), 397–403. 10.1136/gut.47.3.397 10940278 PMC1728045

[B94] Serino M. (2019). SCFAs - the Thin Microbial Metabolic Line Between Good and Bad. Nat. Rev. Endocrinol. 15 (6), 318–319. 10.1038/s41574-019-0205-7 30976118

[B95] Shamekhi S. Lotfi H. Abdolalizadeh J. Bonabi E. Zarghami N. (2020). An Overview of Yeast Probiotics as Cancer Biotherapeutics: Possible Clinical Application in Colorectal Cancer. Clin. Transl. Oncol. 22 (8), 1227–1239. 10.1007/s12094-019-02270-0 31919760

[B96] Singh R. K. Chang H. W. Yan D. Lee K. M. Ucmak D. Wong K.. (2017). Influence of Diet on the Gut Microbiome and Implications for Human Health. J. Transl. Med. 15 (1), 73. 10.1186/s12967-017-1175-y 28388917 PMC5385025

[B97] Slavin J. (2013). Fiber and Prebiotics: Mechanisms and Health Benefits. Nutrients 5 (4), 1417–1435. 10.3390/nu5041417 23609775 PMC3705355

[B98] Sokol H. Adolph Timon E. (2018). The Microbiota: An Underestimated Actor in Radiation-Induced Lesions? Gut 67, 1–2. 10.1136/gutjnl-2017-314279 28473631

[B99] Somosy Z. Horváth G. Telbisz A. Réz G. Pálfia Z. (2002). Morphological Aspects of Ionizing Radiation Response of Small Intestine. Micron. 33 (2), 167–178. 10.1016/S0968-4328(01)00013-0 11567886

[B100] Spiljar M. Merkler D. Trajkovski M. (2017). The Immune System Bridges the Gut Microbiota With Systemic Energy Homeostasis: Focus on TLRs, Mucosal Barrier, and SCFAs. Front. Immunol. 8, 1353. 10.3389/fimmu.2017.01353 29163467 PMC5670327

[B101] Sureban S. M. May R. Qu D. Chandrakesan P. Weygant N. Ali N.. (2015). Dietary Pectin Increases Intestinal Crypt Stem Cell Survival Following Radiation Injury. PloS One 10 (8), e0135561. 10.1371/journal.pone.0135561 26270561 PMC4536042

[B102] Tedelind S. Westberg F. Kjerrulf M. Vidal A. (2007). Anti-Inflammatory Properties of the Short-Chain Fatty Acids Acetate and Propionate: A Study With Relevance to Inflammatory Bowel Disease. World J. Gastroenterol. 13 (20), 2826–2832. 10.3748/wjg.v13.i20.2826 17569118 PMC4395634

[B103] Teo M. T. Sebag-Montefiore D. Donnellan C. F. (2015). Prevention and Management of Radiation-Induced Late Gastrointestinal Toxicity. Clin. Oncol. (R Coll. Radiol.) 27 (11), 656–667. 10.1016/j.clon.2015.06.010 26129746

[B104] Terzi C. Sevinç A. I. Koçdor H. Oktay G. Alanyali H. Küpelioğlu A.. (2004). Improvement of Colonic Healing by Preoperative Rectal Irrigation With Short-Chain Fatty Acids in Rats Given Radiotherapy. Dis. Colon Rectum. 47 (12), 2184–2194. 10.1007/s10350-004-0724-7 15657672

[B105] Tian T. Zhao Y. Yang Y. Wang T. Jin S. Guo J.. (2020). The Protective Role of Short-Chain Fatty Acids Acting as Signal Molecules in Chemotherapy- or Radiation-Induced Intestinal Inflammation. Am. J. Cancer Res. 10 (11), 3508–3531. 33294252 PMC7716145

[B106] Touchefeu Y. Montassier E. Nieman K. Gastinne T. Potel G. Bruley des Varannes S.. (2014). Systematic Review: The Role of the Gut Microbiota in Chemotherapy- or Radiation-Induced Gastrointestinal Mucositis - Current Evidence and Potential Clinical Applications. Aliment Pharmacol. Ther. 40 (5), 409–421. 10.1111/apt.12878 25040088

[B107] Turnbaugh P. J. Ley R. E. Mahowald M. A. Magrini V. Mardis E. R. Gordon J. I. (2006). An Obesity-Associated Gut Microbiome With Increased Capacity for Energy Harvest. Nature 444 (7122), 1027–1031. 10.1038/nature05414 17183312

[B108] Ursell L. K. Haiser H. J. Van Treuren W. Garg N. Reddivari L. Vanamala J.. (2014). The Intestinal Metabolome: An Intersection Between Microbiota and Host. Gastroenterology 146 (6), 1470–1476. 10.1053/j.gastro.2014.03.001 24631493 PMC4102302

[B109] van de Wetering F. T. Verleye L. Andreyev H. J. Maher J. Vlayen J. Pieters B. R.. (2016). Non-Surgical Interventions for Late Rectal Problems (Proctopathy) of Radiotherapy in People Who Have Received Radiotherapy to the Pelvis. Cochrane Database Syst. Rev. 4, CD003455. 10.1002/14651858.CD003455.pub2 27111831 PMC7173735

[B110] Vernia P. Fracasso P. L. Casale V. Villotti G. Marcheggiano A. Stigliano V.. (2000). Topical Butyrate for Acute Radiation Proctitis: Randomised, Crossover Trial. Lancet 356 (9237), 1232–1235. 10.1016/s0140-6736(00)02787-2 11072942

[B111] Visich K. L. Yeo T. P. (2010). The Prophylactic Use of Probiotics in the Prevention of Radiation Therapy-Induced Diarrhea. Clin. J. Oncol. Nurs. 14 (4), 467–473. 10.1188/10.CJON.467-473 20682502

[B112] Vital M. Howe A. C. Tiedje J. M. (2014). Revealing the Bacterial Butyrate Synthesis Pathways by Analyzing (Meta)Genomic Data. mBio 5 (2), e00889. 10.1128/mBio.00889-14 24757212 PMC3994512

[B113] Wang H. G. Huang X. D. Shen P. Li L. R. Xue H. T. Ji G. Z. (2013). Anticancer Effects of Sodium Butyrate on Hepatocellular Carcinoma Cells *In Vitro* . Int. J. Mol. Med. 31 (4), 967–974. 10.3892/ijmm.2013.1285 23440283

[B114] Wang A. Ling Z. Yang Z. Kiela P. R. Wang T. Wang C.. (2015). Gut Microbial Dysbiosis may Predict Diarrhea and Fatigue in Patients Undergoing Pelvic Cancer Radiotherapy: A Pilot Study. PloS One 10 (5), e0126312. 10.1371/journal.pone.0126312 25955845 PMC4425680

[B115] Wang Z. Wang Q. Wang X. Zhu L. Chen J. Zhang B.. (2019). Gut Microbial Dysbiosis is Associated With Development and Progression of Radiation Enteritis During Pelvic Radiotherapy. J. Cell Mol. Med. 23 (5), 3747–3756. 10.1111/jcmm.14289 30908851 PMC6484301

[B116] Wang Y. Wiesnoski D. H. Helmink B. A. Gopalakrishnan V. Choi K. DuPont H. L.. (2018). Fecal Microbiota Transplantation for Refractory Immune Checkpoint Inhibitor-Associated Colitis. Nat. Med. 24 (12), 1804–1808. 10.1038/s41591-018-0238-9 30420754 PMC6322556

[B117] Wang J. Zheng H. Kulkarni A. Ou X. Hauer-Jensen M. (2006). Regulation of Early and Delayed Radiation Responses in Rat Small Intestine by Capsaicin-Sensitive Nerves. Int. J. Radiat. Oncol. Biol. Phys. 64 (5), 1528–1536. 10.1016/j.ijrobp.2005.12.035 16580503

[B118] Wedlake L. Shaw C. McNair H. Lalji A. Mohammed K. Klopper T.. (2017). Randomized Controlled Trial of Dietary Fiber for the Prevention of Radiation-Induced Gastrointestinal Toxicity During Pelvic Radiotherapy. Am. J. Clin. Nutr. 106 (3), 849–857. 10.3945/ajcn.116.150565 28679552

[B119] Wong M.-L. Inserra A. Lewis M. D. Mastronardi C. A. Leong L. Choo J.. (2016). Inflammasome Signaling Affects Anxiety- and Depressive-Like Behavior and Gut Microbiome Composition. Mol. Psychiatry 21, 797–805. 10.1038/mp.2016.46 27090302 PMC4879188

[B120] Wu X. Zhang T. Chen X. Ji G. Zhang F. (2019). Microbiota Transplantation: Targeting Cancer Treatment. Cancer Lett. 452, 144–151. 10.1016/j.canlet.2019.03.010 30905818

[B121] Xing P. Y. Pettersson S. Kundu P. (2020). Microbial Metabolites and Intestinal Stem Cells Tune Intestinal Homeostasis. Proteomics, 20 (5-6), e1800419. 10.1002/pmic.201800419 31994831

[B122] Yadav M. Verma M. K. Chauhan N. S. (2018). A Review of Metabolic Potential of Human Gut Microbiome in Human Nutrition. Arch. Microbiol. 200 (2), 203–217. 10.1007/s00203-017-1459-x 29188341

[B123] Yang J. Ding C. Dai X. Lv T. Xie T. Zhang T.. (2017). Soluble Dietary Fiber Ameliorates Radiation-Induced Intestinal Epithelial-To-Mesenchymal Transition and Fibrosis. JPEN J. Parenter Enteral Nutr. 41 (8), 1399–1410. 10.1177/0148607116671101 27660288

[B124] Yarnold J. Brotons M. C. (2010). Pathogenetic Mechanisms in Radiation Fibrosis. Radiother Oncol. 97 (1), 149–161. 10.1016/j.radonc.2010.09.002 20888056

[B125] Zhang Y. Zhang B. Dong L. Chang P. (2019). Potential of Omega-3 Polyunsaturated Fatty Acids in Managing Chemotherapy- or Radiotherapy-Related Intestinal Microbial Dysbiosis. Adv. Nutr. 10 (1), 133–147. 10.1093/advances/nmy076 30566596 PMC6370266

